# Community asset participation and social medicine increases qualities of life

**DOI:** 10.1016/j.socscimed.2020.113149

**Published:** 2020-08

**Authors:** Luke A. Munford, Maria Panagioti, Peter Bower, Suzanne M. Skevington

**Affiliations:** a Health Organisation, Policy, and Economics Research Team, Centre for Primary Care & Health Services Research, Manchester Academic Health Science Centre, University of Manchester, Manchester, UK; b NIHR School for Primary Care Research, NIHR Greater Manchester Patient Safety Translational Research Centre, Manchester Academic Health Science Centre, University of Manchester, M13 9PL, UK; c NIHR School for Primary Care Research, Centre for Primary Care, Division of Population of Health, Health Services Research and Primary Care, Manchester Academic Health Science Centre, University of Manchester, Manchester, UK; d Manchester Centre for Health Psychology, Division of Psychological Sciences and Mental Health, Coupland 1 Building, University of Manchester, Oxford Road, Manchester, M13 9PL, UK

**Keywords:** Community assets, Social participation, Quality of life, WHOQOL-BREF, Elderly, Social cohesion

## Abstract

**Rationale:**

Social prescribing to community assets, like social groups, is a current policy goal. As aging adults lead longer, healthier lives, the effects of participating in community assets raises questions about whether subjective quality of life (QoL) improves during participation and on what dimensions.

**Objective:**

The study's goal was to examine the effectiveness of community assets at improving QoL among older people living in the community.

**Method:**

Examining longitudinal survey data which tracked health and wellbeing in older adults living in Salford, UK over 12 months, we first used regressions on community assets to compare the World Health Organization's QoL Assessment (WHOQOL-BREF) domains at baseline for those who already participated in community assets (54%) and with non-participants (46%). Second, we used propensity score matching to compare QoL in an ‘uptake’ group (no initial participation but who participated at 12 months), to those who never participated, and to a ‘cessation’ group who participated initially, but ceased within one year, to those who always participated.

**Results:**

Group comparisons confirmed that participants reported significantly higher QoL on all domains – environmental, psychological, physical, and social QoL – and on 16 predicted facets. After affirming group matching reliability, the strongest results were for the uptake group, with significant improvements in all domains, and in 18 facets. All QoL domains decreased in the cessation group, but overall, the effect was weaker. As predicted from the context, QoL relating to ‘opportunities for recreation and leisure’ showed the greatest effect. Furthermore, QoL increased with uptake, and decreased with cessation.

**Conclusion:**

Policies to improve QoL in later life should be designed not just to promote community assets, but also maintain participation once initiated.

## Highlights

• Social prescribing to community assets such as social groups is now a policy goal.
• Little is known about their effect on participants' quality of life (QoL).
• Community asset participants reported higher QoL on all four domains.
• Participation uptake strongly led to increased QoL.
• Cessation was detrimental to QoL.

## 1 Introduction

There is renewed policy interest in the idea that community and its cohesion is an important determinant of health and quality of life (QoL). For example, the English National Health Service (NHS) recently published a Long Term Plan (NHS England, 2019) where it committed to championing ‘community approaches’. This approach builds heavily on the notion that an individual's situation can be improved by reducing loneliness and increasing social cohesion. Two connected but distinct elements - social prescribing and community assets - are at the heart of community approaches outlined in this plan. Social prescribing is “… a means of enabling [general practitioners] GPs, nurses and other primary care professionals to refer people to a range of local, non-clinical services” (The Kings Fund, 2017). Whereas community assets are “… the collective resources which individuals and communities have at their disposal, which protect against negative health outcomes and promote health status” (McLean, 2011). This can include a variety of non-clinical ‘interventions’ such as leisure, education, and the arts. In their national campaign to tackle loneliness, the UK Royal College of General Practitioners argued that loneliness may be as bad for patients as chronic health conditions (Rimmer, 2018). One key recommendation was to “… encourage everyone to take action to tackle loneliness by […] getting involved with their local community.” (Royal College of General Practitioners, 2018).

Social prescribing emerged from recognizing the vital contribution that communities can make to health and well-being. It may be used as a stand-alone intervention or, more often, as a component of more complex interventions. Social prescribing works by referring people to a variety of non-clinical and non-health assets (Robinson, 2018). It is an innovative model of healthcare because it is patient-centred, provides an effective framework for involving patients in their community, and aligns with national agendas for promoting collaboration between service sectors (PHE and NHS England, 2015). Although different models of social prescribing are developing to meet the diverse needs of communities and individuals, community development is advocated to be its fundamental element in which community assets play an important role (Chatterjee et al., 2018). In contrast to a deficits approach, Lisa McNally, the UK Director of Public Health, argues that “… effective social prescribing requires effective community development. Local people should be supported to develop and maintain initiatives offering social interaction. Importantly, this should be ‘asset based’, and driven by the ideas and experience of local people. Professionals should support and facilitate - not define and direct” (McNally, 2018). One type of asset legislated in the UK by the Localism Act of 2011 was defined as: “… buildings or amenities that play a vital role in local life. They might include community centres, libraries, swimming pools, village shops, markets or pubs.” (House of Commons, 2016).

Recently, Munford et al. (2017) showed that community assets are potentially beneficial to improving health-related QoL, as measured with the EQ5D (Euro-QoL group, 2015). Although the EQ5D is the most frequently used measure of health-related QoL in trials and economic evaluations, it assesses only physical and psychological well-being. Growing evidence indicates that QoL is a broader concept, and that its measurement is incomplete without accounting for elements like perceived capabilities, quality of personal and social relationships, and effective participation (Coast et al., 2008; Couzner et al., 2013; Engel et al., 2016). Capability assessments like the ICECAP for Older people (ICECAP-O; Coast et al., 2008), have been developed to supplement the EQ5D instrument, and are increasingly used in economic evaluations and trials, particularly among older people. The World Health Organization's Quality of Life Assessment (WHOQOL-BREF) is a more comprehensive measure of subjective QoL as applied to health that not only assesses physical and psychological QoL, but also QoL domains on the environment and social relationships (The WHOQOL Group, 1998a,b; Skevington et al., 2004a, Skevington et al., 2004b). Given the aims and scope of activities like social prescribing and their links to diverse community assets, it is clear that QoL enhancements may be gained from a wide range of activities, not just those directly related to health. Using the WHOQOL-BREF as a core outcome measure in evaluations of social prescribing interventions offers a unique advantage, as it assesses 25 QoL dimensions (facets) that are relevant and important to diverse cultures world-wide (Skevington et al., 2004a, Skevington et al., 2004b). Additionally, most of the included QoL facets are not assessed by generic measures, such as the EQ5D. The WHOQOL-BREF instrument will, therefore, be more likely to sensitively capture the full range of benefits from social prescribing interventions and participation in community assets, and hence should provide superior evidence regarding effectiveness and cost-effectiveness in these types of intervention.

Social cohesion and social interactions are associated with decreased depression among older adults in Shanghai (Miao et al., 2019). Elderly Chinese residents living in neighbourhoods of lower socioeconomic status were more likely to interact with neighbours, generating higher social cohesion. Increased social cohesion was then associated with lower rates of depression. In Japan, increased social activity among older adults led to fewer incidences of frailty (Sato et al., 2020).

The current study investigates whether there are broad benefits to QoL from participating in community assets by examining WHOQOL-BREF outcomes. The first aim is to find out whether this intervention is effective in promoting QoL on all its important dimensions. A second aim is to observe whether the WHOQOL-BREF is an appropriate tool for evaluating and monitoring outcomes from social prescribing interventions, specifically community assets. After investigating baseline differences between participation groups in each QoL domain and their individual facet components, we tracked the same cohort of older adults for 12 months to examine whether changes in community asset participation significantly affected QoL. This research is theoretically informed by the World Health Organization's definition of subjective QoL: “An individual's perception of their position in life, in the context of the culture and value systems in which they live, and in relation to their goals, expectations, standards and concerns” (The WHOQOL Group, 1995).

## 2 Method

### 2.1 Data

Data taken from the UK National Institute of Health Research funded Comprehensive Longitudinal Assessment of Salford Integrated Care (CLASSIC) study is described elsewhere (e.g., Bower, 2018; Munford et al., 2017). Questionnaires were mailed to older adults aged 65 years or older, who had one or more long-term health conditions diagnosed by a GP. The study population was drawn from disease registers in 33 General Practices within Salford (in urban north-west England). Baseline questionnaires were followed up with postal surveys at six, 12, and 18 months. The research focuses on data collected at baseline, during the winter of 2014 and 2015, and the 12-month follow-up.

An initial baseline sample consisted of 3686 adults over 65 years of age. At the 12-month follow-up, 2820 participants (77%) remained. A flowchart describing the reasons for data loss at various stages appears in Figures A1 and A2 (in the Supplementary Appendix).

### 2.2 Measures of interest

#### 2.2.1 The WHOQOL-BREF short-form

The WHOQOL-BREF is an international, generic patient-reported outcome measure (PROM) developed by a collaboration of 15 cultures through the World Health Organization that was designed for both health and sick people to self-report their QoL (The WHOQOL Group, 1998a,b; Skevington et al., 2004). Focus groups of patients, health professionals, and community members in 15 cultures contributed to an international pool of concepts, contents, and language. The WHOQOL-BREF short-form measure has been internationally standardized, is reliable and valid (The WHOQOL Group, 1998a,b; Skevington et al., 2004), and responds to change in clinical and social conditions (Skevington and Epton, 2018). Five-point interval scales were developed (Szabo et al., 1996; Skevington and Tucker, 1999) to assess the 25 dimensions. One facet assesses general overall QoL and health (two items). Twenty-four items represent specific facets of QoL and are scored in one of four domains: physical, psychological, social relations, and environment. Raw domain scores are transformed onto a 0–100 scale to permit comparisons. Negatively phrased items are reverse scored, so that high scores consistently indicate good QoL. Cases are deemed unreliable when 20% or more responses are missing, or when two or more items are missing from a domain (one for social). The WHOQOL-BREF was collected at baseline and a 12-month follow-up. In UK, the measure is widely used in clinical trials and community populations (Skevington and McCrate, 2012), and is acceptable to adults, including older people (The WHOQOL-OLD Group, 2006).

#### 2.2.2 Community asset participation

Community asset participation was defined using a binary classification (yes or no) to create an indicator variable that takes the value 1 when any community asset is used, and 0 otherwise. Individuals were shown seven listed community assets (see Table 1) and invited to tick all that apply. We focused on the binary indicator variable, as the way individuals self-report the same asset was heterogeneous. For instance, during discussions with users of a particular community asset (‘Tea and Tech’, where members were shown how to use computing facilities while drinking tea and chatting with friends) revealed that some reported this asset as ‘Group for elderly or older people’; some perceived it as ‘Education, arts, music or singing group’, and others classified it as ‘Other group or organization’. We also found evidence of some resistance to reporting themselves as belonging to a ‘Group for elderly or older people’. This binary classification helps to overcome different reports of the same asset.

**Table 1:** Rates of community asset participation over time.

	Baseline (%)	12 months (%)
**Participation in community assets**	54	59
*Type of asset:*		
Group for elderly or older people (e.g. lunch club)	11	12
Education, arts, music or singing group (including evening classes)	8	9
Religious group or church organisation	20	20
Charity, voluntary or community group	15	14
Social club (including Working Men's Clubs, Rotary Clubs, etc.)	14	18
Sports club, gym, exercise, or dance group	21	23
Other group or organisation	18	20
**I don't regularly join in any of the activities of these organisations**	46	41

*Note*. Based on the fixed sample of *N* = 2820 individuals included in the primary analysis. Numbers sum to more than 100% as respondents could check more than one option.

For similar reasons, we do not consider the intensity of participation by looking at the count of assets reported. During discussions, some individuals reported that they indicated the same community asset (‘Tea and Tech’) as fitting a number of categories, and hence, ticked them all. Therefore, we could over-estimate the true number of assets used if we added up the number of assets ticked. Information on community asset participation was collected twice, at baseline and follow-up.

#### 2.2.3 Sociodemographic and socioeconomic characteristics

In the analysis, we controlled for the variables of gender (women or men) and age (split into five-year bands; ranging from 65 to 69 years - the reference age group - up to 85 or older). Living situation was recoded into categories of ‘with spouse’, ‘with other’, or ‘alone’ (reference category). Coding categories of qualifications allowed multiple responses: ‘no qualifications’ (reference); ‘one or more Ordinary ‘O’ Level certificates; Certificate of Secondary Education (CSEs or GCSEs), ‘one or more Advanced Level certificates (A/AS-Levels), ‘Degree’, ‘National Vocational Qualification (NVQ)’, ‘Trade qualification’, and ‘Professional qualification’. These educational classifications are standard, particularly in the UK, but it is worth noting there is no strict hierarchy among them. Sociodemographic and socioeconomic variables were only reported at baseline.

#### 2.2.4 Healthcare utilization and the presence of limiting health conditions

Respondents were matched to their administrative health records using their NHS Number. This allowed us to construct detailed information on use of primary and secondary health services in the six months prior to baseline. Individual respondent-level healthcare resource utilization over the study period was collected from two sources: the number of GP contacts in the previous six months on electronic primary care databases, and hospital utilization information which was extracted from linked administrative patient records provided by the NHS. The second category of data was divided into emergency admissions (short stays ≤ five days or long stays > five days), elective admissions, elective day cases, outpatient attendances, and accident and emergency (A&E) department attendances (following Panagioti et al., 2018).

Individuals were also shown a list of 23 common long-term health conditions to indicate how much each condition limited their daily activity on a six-point (0–5) Bayliss score. We recoded scores of four or five to indicate that the individual's condition was limiting, whereas scores of three or lower indicated non-limiting conditions (Munford et al., 2017). We repeated this scoring for all 23 conditions, as an individual could have more than one limiting health condition. We did not consider the count of these limiting conditions, rather whether or not an individual had the condition and it limited their daily activities.

### 2.3 Statistical analysis

#### 2.3.1 Mean differences

Mean transformed baseline scores were calculated for domains by community asset participation status to obtain QoL means for different participation groups. Using a paired students t-test for unequal groups, statistical differences for participation groups were compared at baseline and at the 12-month follow-up. The effect size (mean difference divided by the pooled standard deviation) was computed.

#### 2.3.2 Baseline regressions

Simply examining differences in mean outcome scores is problematic, as they may be confounded. For instance, if non-participants are much older or younger than participants, their scores could differ due to age, rather than participation. To control for this factor, we exploited the cross-sectional nature of the baseline survey and regressed domain scores against the same series of covariates known to influence them. To ease interpretation, all outcomes were standardized using the z-score (an individual's response minus the average response, all divided by the standard deviation).

We use cross-sectional baseline only data, as only this point reported the potential confounding variables. The analysis is performed on 3686 individuals with useable baseline data, and is not dependent on remaining in the sample. The model of interest was:

(1) $$y_{i}=\alpha +\beta CA_{i}+\gamma X_{i}+\delta H_{i}+\varepsilon_{i}$$

In this model, *y* is the standardized outcome (WHOQOL-BREF domain scores or individual facet response), *CA* is a binary variable (coded as a 1 if the individual, *i*, participated in community assets or 0 if the individual, *i* was a non-participant), *X* is a vector of socioeconomic and sociodemographic information (gender, age bands, educational qualifications, and living arrangements), and *H* is a vector of health information (whether individual, *i* has any one of 23 limiting health conditions, and their healthcare utilization six months’ prior to baseline).

#### 2.3.3 Longitudinal models: propensity score matching (PSM)

In a single cross-section of baseline data, there are problems with the possible bi-directional nature of the relationship between community asset participation and QoL.

As any observable difference in outcomes might drive the participation decision, we cannot be sure whether community asset participants have better QoL scores or conversely, those with better QoL are more likely to participate. The current study aimed to investigate the effects of participation in community assets on an individual's QoL. To do this, we exploited the longitudinal nature of the data, and made two sets of comparisons based on prospective case-control analysis:

1. To understand the potential benefits of community asset uptake, we compared individuals who did not participate in community assets at baseline, but did start to participate before the 12-month follow-up (No-Yes, “NY”: the ‘case’ or ‘treatment’ group), with individuals who never participated in community assets at any time during the study (No-No, “NN”: the ‘control group’). This was the ‘uptake’ case.
2. To understand the potential disadvantages of community asset cessation, we compared individuals who did participate in community assets at baseline, but stopped participating before the 12-month follow-up (Yes-No “YN”: the ‘case’ or ‘treatment’ group), with individuals who always participated in community assets throughout the study (Yes-Yes “YY”: the ‘control’ group). This was the ‘cessation’ case.

We argue that it is important to look at both sides, as *a priori,* it is unclear whether there would be symmetrical effects for uptake and cessation.

To overcome the issues above, we used PSM, which is defined as the probability of being assigned to a given treatment – in this case, decisions around community asset participation – given a set of observed covariates. PSM is useful when treatment is not randomly assigned, but a choice. Introduced by Rosenbaum and Rubin (1983), it identifies ‘neighbours’ that are as similar as possible to each other with respect to receiving the treatment (i.e., starting or stopping participation in community assets). Focusing on the uptake case, we took an individual at baseline who did not participate in community assets, but did so after 12 months, and matched them to a participant who was as similar as possible in terms of socio-demographics and health, but still did not participate in community assets within one year. Consequently, we compared QoL in these two subgroups at 12 months, as the only observable difference between the matched individuals was the decision to participate in community assets. The underlying assumption is that the matched individual can be considered as a counterfactual for the individual who started to participate.

Propensity scores were calculated using logistic regression where the outcome is a binary variable for having started to participate by the 12-month follow-up. This is regressed on a set of observed covariates reported at baseline, which are used as matching variables, namely: age (year bands), gender, living arrangements (living alone, with spouse, or with another person), EQ5D utility score, the four WHOQOL-BREF domain scores, and highest educational level attained (qualifications at school, college, university, NVQ or trade, and professional). In robustness checks, we varied the variable included in the matching algorithm, and the results are robust. For example, EQ5D scores were removed, as these could potentially be a direct determinant of some WHOQOL-BREF domains, and duplicated QoL information would inflate the findings. In this PSM analysis, paired treated and control individuals are identified by having a similar propensity score, which is pre-defined by a maximum acceptability range known also as calliper width. We used PSM with replacement, which means that individuals could be used as matched-controls for multiple treated individuals. The strength of PSM techniques were evaluated by comparing means for treated and control individuals before and after matching, as well as reporting the distribution of the propensity score after matching for treated and control individuals analyzed.

Once the treatment group was matched with similar individuals in the (matched) control group, the effect size was the Average Treatment Effect (ATE), which is defined as the differences in outcomes between the two groups. Given that matching should ensure that both groups are as similar as possible on observable characteristics, the ATE can be interpreted as the effect of community asset participation or cessation on the outcomes.

#### 2.3.4 Predictions

The following QoL features were expected to facilitate meeting others while accessing community assets. It was predicted from the literature that, of the 26 WHOQOL-BREF items, 16 (in 15 facets), would be significantly, positively associated with participation. Distinctive profiles of selected QoL information about participants and non-participants could later be useful to health and social care professionals who seek to promote uptake.

From the study context, it was predicted that if participants reported higher QoL from perceiving more opportunities for *leisure and recreation*, confirmation would also add validity to this facet. Within the environment domain, participants were also expected to perceive better QoL from their *physical environment*, more *physical safety and security*, and greater opportunities to access *health and social care*. The expectation of better QoL in these environmental facets could facilitate leaving home to attend community events.

Better social relationships QoL derived from *personal relationships* and *social* support was expected for participants, as such qualities could actively promote communications during participation. From the psychological domain, it was predicted that more *positive feelings*, higher *self-esteem*, better *body image*, and less *negative feelings* would enhance encounters with others at community events. For the physical domain, participants were expected to derive better QoL from more *energy, mobility,* and *sleep*, and less *pain and discomfort*, as such features could improve their capacity to physically attend, and actively participate in community assets. Lastly, participants were expected to report better overall *general QoL*, and more *subjective health*.

#### 2.3.5 Imputation of missing data and multiple hypothesis testing

This analysis is based on complete-case data, as we did not use imputation strategies. We did this as the key ‘treatment’ variable. Whether or not an individual participated in community assets was not randomly assigned, but was a choice made by the individual. We therefore did not feel we could meaningfully impute this variable.

Of the 691 individuals who returned a questionnaire at baseline, but were omitted from the analysis, 667 (96.5%) had no information on community asset participation. One of the possible options was “I don't regularly join in any of the activities of these organisations”, and this was used as the ‘non-participant’ group. However, in a robustness check (not presented in this paper), we have included the individuals with missing information on community asset participation status as ‘non-participants’, and the results remain qualitatively very similar. However, we do not present these results, as we believe that assuming that all individuals who do not answer the section on “Community Assets” are non-participants, is an overly strong assumption.

Likewise, at the 12-month follow-up of the 570 individuals who returned a questionnaire, but were omitted from the longitudinal analysis, 536 (94%) had no information on community asset participation. However, all of these individuals also did not report any community asset participation information at baseline, so it is highly likely they are in the ‘never participate’ group. Again in a robustness check, we include these individuals in the “No-No” group, and the results remain qualitatively the same. However, we do not present this analysis, as it is based on the strong assumption that missing information is indicative of non-participation.

The main results presented do not account for multiple hypothesis testing. We acknowledge that we simultaneously test multiple hypotheses for the many outcome measures we consider, but do not apply the Bonferroni correction, which essentially entails dividing the standard p-value (*p* = 0.05) by the number of comparisons made. Yet the reasons we do not do so are two-fold: First, our predictions are based on pre-specified hypotheses (see Section 2.3.4). Before any analysis was conducted, we hypothesised which domains, and which facets we expected to be affected, and why. Second, the typical multiple hypothesis testing literature does not account for the fact that some of our outcomes (e.g., the domain scores) are formed from responses to the individual facets. The Bonferroni correction ignores that nuance, and could lead to the incorrect rejection of some null hypotheses.

## 3 Results

### 3.1 Summary statistics

Table 2 presents selected summary statistics of key variables for the complete case sample. Outcome variables are available for both time points, whereas confounders (and matching variables) are only recoded once. At baseline, 1523 individuals (54%) reported that they had participated in one or more community assets, compared to 1297 (46%) who did not participate in any community asset over the six months prior to the study. The participant numbers increased to 1664 (59%) at the 12-month follow-up. Table 1 presents a more detailed breakdown of the types of asset use across the two periods. At baseline, mean WHOQOL-BREF domain scores were consistently higher among participants than non-participants, and all results were strongly significant. For example, participants had a mean physical domain score of 65.80 indicating good QoL, compared to 57.99 for non-participants, indicating QoL was neither good nor poor (difference 7.82; 95% *CI*: 6.28 to 9.35). The effect size of asset participation as assessed by the difference in the standardised domain scores, is largest for environmental QoL (effect size: 0.45; 95% *CI*: 0.39 to 0.52), then physical QoL (effect size: 0.35; 95% *CI*: 0.28 to 0.42) and psychological QoL (effect size: 0.32; 95% *CI*: 0.25 to 0.39) domains. The social domain showed the smallest effect size (0.22; 95% *CI*: 0.15 to 0.29). The mean level of sample QoL was very good (>70.0) across psychological, social, and environmental domains.

**Table 2:** Selected summary statistics of key variables.

Time period:	Baseline			12-month follow-up		
Participate in CAs:	Yes	No	Difference^a^ (95% *CI*)	Yes	No	Difference^a^ (95% *CI*)
*N* (%)	1523 (54%)	1297 (46%)		1664 (59%)	1156 (41%)	
*Outcomes*						
Physical domain	65.80 (19.86)	57.99 (22.80)	7.82 (6.28–9.35)	63.88 (18.72)	55.74 (22.30)	8.15 (6.69–9.60)
(standardised)	0.26 (0.89)	−0.09 (1.02)	0.35 (0.28–0.42)	0.17 (0.90)	−0.22 (1.07)	0.39 (0.32–0.46)
Psychological domain	73.88 (14.80)	68.23 (18.27)	5.65 (4.46–6.84)	71.15 (14.77)	65.02 (18.47)	6.13 (4.96–7.30)
(standardised)	0.24 (0.84)	−0.08 (1.03)	0.32 (0.25–0.39)	0.16 (0.88)	−0.20 (1.10)	0.37 (0.30–0.43)
Social relations domain	71.45 (18.91)	66.98 (20.42)	4.46 (3.04–5.89)	69.24 (18.46)	65.01 (20.50)	4.23 (2.84–5.62)
(standardised)	0.15 (0.94)	−0.07 (1.01)	0.22 (0.15–0.29)	0.10 (0.95)	−0.12 (1.05)	0.22 (0.15–0.29)
Environmental domain	77.54 (14.41)	70.10 (16.46)	7.44 (6.33–8.55)	75.27 (14.60)	68.10 (16.58)	7.17 (6.09–8.26)
(standardised)	0.31 (0.88)	−0.15 (1.00)	0.45 (0.39–0.52)	0.20 (0.90)	−0.25 (1.06)	0.46 (0.39–0.53)
*Confounders/matching*						
Female	0.52	0.51	0.02 (−0.15 to 0.05)	NA		
Aged 65–69 years	0.30	0.30	0.00 (−0.03 to 0.03)	NA		
Aged 70–74 years	0.28	0.27	0.02 (−0.01 to 0.05)	NA		
Aged 75–79 years	0.22	0.21	0.01 (−0.02 to 0.04)	NA		
Aged 80–84 years	0.12	0.13	−0.01 (−0.03 to 0.02)	NA		
Aged 85+ years	0.07	0.09	−0.02 (−0.04 to 0.01)	NA		
Live alone	0.34	0.36	−0002 (−0.05 to 0.02)	NA		
Live with spouse	0.60	0.57	0.03 (0.00–0.70)	NA		
Live with other	0.10	0.12	−0.02 (−0.04 to 0.01)	NA		
EQ5D score	0.78 (0.21)	0.71 (0.26)	0.08 (0.06–0.09)	NA		

*Note.* Mean values (standard deviations) are reported. *N* = 2820.

a Differences calculated using an independent two-sample *t*-test, accounting for unequal sample sizes.

When considering confounders, there is very little evidence of differences between those who do and those who do not participate in community assets at baseline. For example, 52% of participants are female, compared to 51% of non-participants. The age distribution was very similar, as were living arrangements.

### 3.2 Baseline regressions

Table 2 indicated that, on average, participants reported higher baseline WHOQOL-BREF scores than non-participants, but again, this could be due to unobservable differences. We next present regression results of baseline WHOQOL-BREF scores on known confounders. Table 3 reports the coefficient (known as *β*) on community asset participation, from a regression of community asset participation on scores for each of four standardized QoL domains, and accounts for factors known to be correlated with them (see Equation (1) and Table 3 notes).

**Table 3:** The effect of community asset participation on transformed WHOQOL-BREF domain scores and each item at baseline.

Panel and variable	Coefficient (β)	95% *CI*	*p*	*R*^*2*^
**Panel (a): Standardised domain scores**				
*Physical domain*	0.185***	[0.134,0.236]	*p* < 0.001	0.45
*Psychological domain*	0.225***	[0.166,0.285]	*p* < 0.001	0.24
*Social relations domain*	0.167***	[0.103,0.231]	*p* < 0.001	0.21
*Environmental domain*	0.318***	[0.259,0.377]	*p* < 0.001	0.24
**Panel (b): Standardised WHOQOL-BREF items**				
*General Quality of Life^*	0.282***	[0.224,0.340]	*p* < 0.001	0.26
*Overall Health^*	0.164***	[0.106,0.223]	*p* < 0.001	0.25
*Pain and discomfort*^*#^*^	0.060*	[0.006,0.113]	*p* = 0.028	0.38
*Dependence on treatment*^*#*^	0.050	[-0.010,0.111,]	*p* = 0.103	0.21
*Positive feelings^*	0.303***	[0.241,0.364]	*p* < 0.001	0.16
*Spiritual Quality of Life*	0.224***	[0.160,0.288]	*p* < 0.001	0.12
*Cognitions (e.g. concentration)*	0.051	[-0.013,0.114]	*p* = 0.119	0.11
*Physical safety and security^*	0.140***	[0.078,0.203]	*p* < 0.001	0.15
*Physical environment^*	0.223***	[0.160,0.287]	*p* < 0.001	0.17
*Energy and fatigue^*	0.174***	[0.118,0.231]	*p* < 0.001	0.29
*Body Image and appearance^*	0.147***	[0.085,0.208]	*p* < 0.001	0.16
*Financial resources*	0.184***	[0.122,0.247]	*p* < 0.001	0.14
*Access to Information and skills*	0.168***	[0.105,0.232]	*p* < 0.001	0.12
*Access to Leisure and recreation^*	0.489***	[0.432,0.546]	*p* < 0.001	0.28
*Mobility^*	0.206 ***	[0.155,0.258]	*p* < 0.001	0.41
*Sleep and rest^*	0.118***	[0.055,0.182]	*p* < 0.001	0.11
*Activities of daily living*	0.196***	[0.141,0.251]	*p* < 0.001	0.33
*Working capacity*	0.203***	[0.146,0.260]	*p* < 0.001	0.34
*Self-esteem^*	0.194***	[0.134,0.254]	*p* < 0.001	0.22
*Personal relations^*	0.094**	[0.031,0.158]	*p* = 0.004	0.13
*Sex-life*	0.098**	[0.028,0.168]	*p* = 0.006	0.13
*Social support^*	0.193***	[0.127,0.258]	*p* < 0.001	0.15
*Home environment*	0.139***	[0.075,0.204]	*p* < 0.001	0.16
*Access to Health and social care^*	0.102**	[0.037,0.167]	*p* = 0.002	0.16
*Transport*	0.161***	[0.099,0.224]	*p* < 0.001	0.13
*Negative feelings*^*#^*^	0.117***	[-0.056,0.179]	*p* < 0.001	0.14

*Note.* All models are estimated separately and the coefficient on community asset participation is shown (β). The outcomes have been standardised by transforming into z-scores. All models additionally control for gender, age (in five-year age bands), educational qualifications, living arrangements, if an individual reports having any one of 23 limiting health conditions (4 or 5 on the Bayliss scale), and the numbers of visits in the 6 months prior to interview for: elective hospital admissions, emergency long stay and short stay hospital visits, day-case hospital visits, outpatient appointments, and visits to accident and emergency (A&E).

Items in *italics* indicate where predicted significance between participants and non-participants was confirmed*.*

^#^ These items are reversed coded, so that positive values indicate higher levels of QoL. For example, a positive value on ‘pain’ indicates that QoL is better for those reporting lower pain. In each facet, high scores mean good QoL.

^ Better QoL predicted for participants and confirmed.

*p < 0.05, **p < 0.01, ***p < 0.001. *N* = 3686 adults aged over 65 years of age.

Table 3 (Panel A) shows that the effect of community asset participation on QoL is positive, large, and significant. Participants report physical domain scores which are 0.185 (standardized) points higher than non-participants (95% *CI*: 0.134 to 0.236), and psychological scores 0.225 points higher (95% *CI*: 0.166 to 0.285). Social domain scores are 0.167 standardized points higher for participants compared to non-participants (95% *CI*: 0.103 to 0.231), and 0.318 points higher in environmental QoL (95% *CI*: 0.259 to 0.377). For participants, the largest gains to QoL are in the environment domain, as predicted, followed by psychological, physical, and social domains.

In Panel B of Table 3, each WHOQOL-BREF item is separately regressed on community asset participation. Expected results are confirmed as the size, significance, and direction of all 16 predicted QoL items, showing that they distinguish between participants and non-participants. For general overall QoL, participants scored 0.282 higher than non-participants (95% *CI*: 0.224 to 0.340) and 0.164 points higher for self-reported health (95% *CI*: 0.106 to 0.223). As predicted from the setting, the largest difference between groups is confirmed for QoL from opportunities to access leisure and recreation, where participants scored 0.489 higher than non-participants (95% *CI* 0.432 to 0.546).

Among environmental QoL facets it was confirmed that compared to non-participants, participants held more positive perceptions of their physical environment (effect size 0.223; 95% *CI*: 0.160 to 0.287), felt more physically safe and secure (effect size 0.140; 95% *CI*: 0.078 to 0.203), and perceived more opportunities to access health and social care (effect size 0.102; 95% *CI*: 0.037 to 0.167).

We also confirmed that greater social QoL was derived from better personal relationships (effect size 0.094; 95% *CI*: 0.031 to 0.158), and social support (effect size 0.193; 95% *CI*: 0.127 to 0.258).

QoL related to mood is better for participants; they reported higher QoL from positive feelings, scoring 0.303 higher than non-participants (95% *CI*: 0.241 to 0.364) and better QoL from fewer negative feelings than non-participants (effect size 0.117; 95% *CI*: 0.056 to 0.179). Among psychological dimensions other than mood, higher QoL was derived from self-esteem (effect size 0.194; 95% *CI*: 0.134 to 0.254), and body image and appearance (effect size 0.147; 95% *CI*: 0.085 to 0.208).

Compared to non-participants, participants reported higher physical QoL relating to more energy (effect size 0.174; 95% *CI*: 0.118 to 0.231), better sleep (effect size 0.118; 95% *CI*: 0.055 to 0.182), greater mobility (effect size 0.206; 95% *CI*: 0.155 to 0.258), and less pain/discomfort (effect size 0.060; 95% *CI*: 0.006 to 0.113).

Examining the remaining 10 QoL facets where group differences were not predicted, eight were significant. These eight significant, non-predicted, factors included spiritual QoL, financial resources, access to information and skills, activities of daily living, working capacity, sex-life, home environment, and satisfaction with their local transport. The two exceptions were facets relating to dependence on medication and treatment, and cognitions (thinking, learning, memory and concentration). In summary, the WHOQOL-BREF distinguishes between participants and non-participants on all four domains, and on 24 out of 26 component items (i.e., 23 QoL facets). The *R*^2^ measures of goodness of fit are all larger than conventional levels, and hence, we conclude the models fit the data well.

### 3.3 Longitudinal models

#### 3.3.1 Reliability test and attrition

**Fig. 1:**
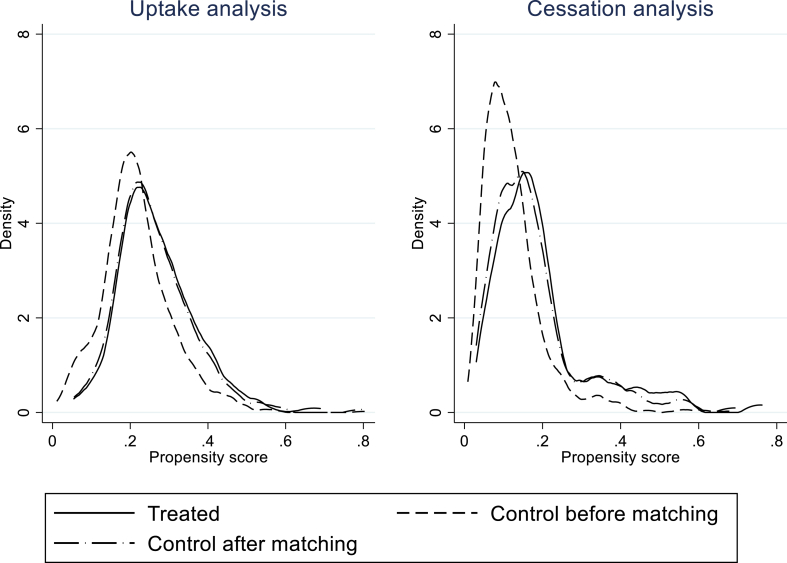
Density plots of propensity scores before and after matching.

The reliability of the results depends on the quality of the propensity score matching (i.e., the comparability of treatment and control groups). Consequently, we tested the average scores of each of the matching variables for treatment and control groups, and observed no evidence that these groups differed after matching (Table A1, Supplementary Appendix). For example, the mean age of the treatment group is 74.12 years and for matched controls is 73.97, implying a difference of 0.15 years (*p* = 0.79). To further graphically test this, the density plots of the propensity scores in the treatment and control groups are shown in Fig. 1. This provides assurance that based on observable factors, the treatment and control groups were much more comparable after matching, and improves confidence in the average treatment effects (ATEs) reported in Table 4.

**Table 4:** The effects of community asset participation uptake and cessation at 12 months on WHOQOL-BREF domain scores and individual items.

Panel and variable	Uptake			Cessation		
	ATE	*95% CI*	*p*	ATE	*95% CI*	*p*
**Panel (a): Standardised domain scores**						
*Physical domain*	0.224***	[0.121,0.328]	p < 0.001	−0.136*	[-0.245,-0.026]	p = 0.015
*Psychological domain*	0.159**	[0.049,0.269]	p = 0.004	−0.051*	[-0.101.-0.001]	p = 0.045
*Social relations domain*	0.111*	[0.004,0.217]	p = 0.041	−0.105*	[-0.193,-0.018]	p = 0.018
*Environmental domain*	0.019*	[0.002,0.036]	p = 0.032	−0.171*	[-0.312,-0.030]	p = 0.017
**Panel (b): WHOQOL-BREF items**						
*General Quality of Life*	0.119**	[0.030,0.209]	p = 0.009	−0.215**	[-0.376,-0.053]	p = 0.009
*Overall Health*	0.178**	[0.047,0.309]	p = 0.008	−0.207*	[-0.393,-0.021]	p = 0.029
*Pain*^*#*^	0.035	[-0.105,0.174]	p = 0.624	0.150	[-0.038,0.338]	p = 0.118
Dependence on treatment ^*#*^	0.132*	[0.005,0.259,]	p = 0.041	0.144	[-0.062,0.351]	p = 0.171
*Positive feelings*	0.198**	[0.071,0.326]	p = 0.002	−0.179*	[-0.343,-0.015]	p = 0.032
Spiritual Quality of Life	0.105*	[0.023,0.186]	p = 0.014	−0.115	[-0.281,0.050]	p = 0.171
Cognitions	0.128*	[0.001,0.255]	p = 0.048	−0.009	[-0.167,0.149]	p = 0.908
*Physical safety*	0.082	[-0.057,0.221]	p = 0.247	−0.096	[-0.255,0.064]	p = 0.240
*Physical environment*	0.150*	[0.025,0.275]	p = 0.019	−0.209	[-0.441,0.024]	p = 0.078
*Energy*	0.279***	[0.147,0.410]	p < 0.001	−0.237**	[-0.403,-0.072]	p = 0.005
*Body image*	0.271***	[0.128,0.413]	p < 0.001	−0.203*	[-0.386,-0.019]	p = 0.030
Financial resources	0.016	[-0.159,0.190]	p = 0.861	−0.068	[-0.248,0.112]	p = 0.459
Access to Information	0.111	[-0.029,0.252]	p = 0.121	−0.195	[-0.397,0.007]	p = 0.059
*Access to Leisure*	0.412***	[0.266,0.558]	p < 0.001	−0.545***	[-0.757,-0.334]	p < 0.001
*Mobility*	0.218**	[0.064,0.372]	p = 0.005	−0.338**	[-0.556,-0.121]	p = 0.002
*Sleep*	0.210**	[0.056,0.364]	p = 0.007	−0.187*	[-0.361,-0.013]	p = 0.035
Activities of daily living	0.172*	[0.021,0.323]	p = 0.025	−0.111*	[-0.218,-0.004]	p = 0.041
Working capacity	0.230**	[0.092,0.368]	p = 0.001	−0.071	[-0-.277,0.135]	p = 0.500
*Self-esteem*	0.156*	[0.026,0.290]	p = 0.019	−0.157*	[-0.308,-0.005]	p = 0.043
*Personal relations*	0.094*	[0.014,0.173]	p = 0.021	−0.191*	[-0.365,-0.016]	p = 0.032
Sex-life	−0.085	[-0.235,0.065]	p = 0.266	−0.006	[-0.213,0.200]	p = 0.951
*Social support*	0.151*	[0.007,0.295]	p = 0.040	−0.192*	[-0.365,-0.018]	p = 0.031
Home environment	−0.065	[-0.211,0.081]	p = 0.381	−0.014	[-0.230,0.201]	p = 0.897
*Access to Health and social care*	0.084	[-0.068,0.236]	p = 0.277	−0.098	[-0.281,0.084]	p = 0.291
Transport	0.136*	[0.003,0.269]	p = 0.045	−0.139	[-0.364,0.087]	p = 0.229
*Negative feelings*^*#*^	0.144*	[0.010,0.279,]	p = 0.035	0.151*	[0.003,0.298]	p = 0.045

*Note*. The average treatment effects (ATEs) are calculated using propensity score matching techniques. The matching variables are listed in Table 3 and additionally include baseline EQ5D scores and baseline outcome variable scores. The uptake results are defined as the difference in scores between those who start to use community assets and those who continue not to use. The cessation results are defined as the difference in scores between those who stop participating in community assets and those who continue to do so.

^#^ These items have been reversed coded, so that positive values indicate higher levels of QoL from lower levels QOL the items. For example, a positive value on ‘pain’ indicates that QoL is better for individuals who report lower levels of pain.

*p < 0.05, **p < 0.01, ***p < 0.001. Items in *italics* indicate where significance was predicted*.*

To examine if initial community asset participation was associated with sample attrition (whether or not an individual remained in the sample up to the 12-month follow-up), we ran a logistic model of attrition (1 was coded if an individual drops-out, 0 if they remain in the sample) as a function of baseline characteristics including QoL, health, and community asset participation. We interacted baseline community asset participation with all of the covariates to see if there were differential associations between attrition and the covariates between those who did or did not participate in community assets at baseline. To ease interpretation, we present odds ratios (ORs) in Table A2 (Supplementary Appendix).

Significant predictors of drop-out from the cohort were EQ5D, older age, education, and having limiting conditions of asthma, bronchitis and osteoporosis. Older people were more likely to drop-out, as were people with lower EQ5D levels. Bronchitis and osteoporosis increased the probability of attrition, but having limiting asthma made it more likely that an individual would remain in the sample.

However, the magnitude of their effects on drop-out were not significantly different between those who initially participated, and those who initially did not participate in community assets. From this we conclude that the main effect of interest, namely the choice to participate, does not affect the probability of attrition either on its own (OR = 1.12; 95% *CI*: 0.36 to 3.43), or interacted with any other health, QoL, or demographic characteristic.

Given the interactions with community asset participation are always statistically insignificant, we conclude we do not have a systematic problem of attrition related to our key effect.

#### 3.3.2 PSM results

Next, we examine the impact of uptake and cessation of participation using propensity score techniques. For the uptake analysis, of the 1297 (46%) individuals who did not participate in community assets at baseline, 342 individuals (26% of 1297) did participate by follow-up. For the cessation analysis, of the 1523 (54%) who participated at baseline, 201 individuals (13%) did not participate after one year. Of the 3686 individuals analyzed at baseline, 2820 remained in the sample at 12-months (77%).

In Table 4, we show that uptake of community assets leads to higher QoL in all domains: physical (ATE: 0.224; 95% *CI*: 0.121 to 0.328); psychological (ATE: 0.159; 95% *CI*: 0.049 to 0.269), social (ATE: 0.111; 95% *CI*: 0.004 to 0.217), and environmental QoL (ATE: 0.019; 95% *CI*: 0.002 to 0.036). These results indicate positive benefits from uptake across all important QoL domains for those who began to participate, compared to similar individuals who continued non-participation. Nineteen items representing 18 facets were significant in Table 4; nine *p* < 0.01. Uptake of community assets is also associated with significant increases in general QoL of 0.119 (95% *CI*: 0.030 to 0.209), and better subjective health, as shown by changes of 0.178 standardised points (95% *CI*: 0.047 to 0.309).

Among the QoL facets, we confirmed that the biggest effect size was for perceived opportunities to access leisure and recreational activities, where the standardised effect size for uptake was 0.412 (95% *CI*: 0.266 to 0.558). Large, positive, significant effect sizes for uptake were also found in five other predicted facets; body image and appearance, energy and fatigue, positive feelings, mobility, and sleep and rest, but the largest effect size was for working capacity, which was not predicted. These six additional facets are in the physical or psychological domains.

Cessation results at the right-hand side of Table 4, show that individuals who stopped using community assets are disadvantaged by reduced QoL, as similar significant decreases in size were found for all four domains: physical (ATE: -0.136; 95% *CI*: -0.245 to −0.026), psychological (ATE: --0.051; 95% *CI*: -0.101 to −0.001), social (ATE: -0.105; 95% *CI*: -0.193 to −0.018), and environmental QoL (ATE: -0.171; 95% *CI*: -0.312 to −0.030).

Twelve facets (13 items) showed a significant decrease in QoL associated with cessation, but as a set, the results were weaker than for uptake, as only four items were considered significant (*p* < 0.01). Mirroring its uptake trend, perceived opportunities for access to leisure and recreation showed the largest QoL decrease (effect size: -0.545; 95% *CI*: -0.757 to −0.3354). Other predicted physical domain facets on mobility and energy show large effects for cessation (p = 0.01–0.001). Ceasing to participate in community assets is associated with decreased general QoL 0.215 (95% *CI*: 0.053 to 0.376), and a small, significant decrease (0.207) for self-reported health (95% *CI*: 0.021 to 0.393). No unpredicted facets were significant for cessation.

## 4 Discussion

The recently published NHS Long Term plan (2019) placed emphasis on the role of community-centred approaches with a particular focus on social prescribing and community assets. The present research suggests that if the NHS is successful in achieving these aims using these types of intervention then the QoL of those who participate should increase. Consistent associations between community asset participation and QoL at baseline, showed that participants report higher subjective QoL across all four domains, with the largest effect for environmental QoL, and within this, access to leisure and recreation. A large effect for psychological QoL illustrated how good mood from positive feelings is salient for participants, and integral to better general QoL overall. Participant and non-participant comparisons revealed differences in almost all facets (23/25) and covered all domains, although only 16 were predicted. This finding demonstrates that participation has a wide-spread effect across almost all internationally important dimensions of QoL assessed by the profile.

Facets that were associated with higher environmental QoL in the baseline analysis included more positive perceptions of their physical environment, and perceived opportunities to access health and social care. These environmental QoL features could facilitate leaving home to attend community events. We also confirmed that greater social QoL was derived from better personal relationships and social support. Such social characteristics may promote more positive interpersonal communications in participants.

QoL related to mood is better for participants; they reported higher QoL from positive feelings, scoring 0.303 higher than non-participants, and better QoL from fewer negative feelings than non-participants. Among psychological dimensions other than mood, higher QoL was derived from self-esteem, and body image and appearance. A positive self-image would likely promote more engagement with community members. Compared to non-participants, participants reported higher physical QoL relating to more energy, better sleep, greater mobility, and less pain/discomfort. These features could improve the person's physical capacity to attend events.

In a further measure of success, rates of community asset participation increased from 54% at baseline, to 59% 12 months later due to 342 individuals starting to participate; this compared with 201 individuals who ceased participation during the same period. The distinctive profiles of selected QoL facets associated with being a participant or non-participant could assist health and social care professionals identify adults over 60-years old who would benefit most from their intervention, to encourage them to take part in attractive community assets. The English language WHOQOL-BREF can be completed in as little as 6 min. These valuable findings confirm the effectiveness of an additional, simple and relatively low-cost intervention, together with an appropriate tool for its long-term monitoring and evaluation. Social prescribing interventions (The Kings Fund, 2017) based on offering community assets to older adults can supplement and enhance the delivery of community medicine, primary care and public health, especially in deprived areas.

We matched propensity scores of individuals who started participating, with similar individuals at baseline who had never participated, to elicit the effects of starting to participate. Uptake led to increased QoL on all domains, showing not just a particularly strong effect on physical QoL, but also a breadth of influence across psychological, social and environmental QoL. Uptake showed more dimensions with large effect sizes than cessation, and from a wider facet range. Subjective qualities of life were more strongly influenced by community asset uptake than the converse trend from cessation.

Although these effects were significant, they followed the opposite pattern in magnitude when compared to baseline analyses, so highlighting the importance of examining longitudinal models, not just cross-sectional associations. When considering separate facets, access to leisure still showed the biggest effect of community asset uptake as expected from the context, and type of intervention studied. At a measurement level, this result adds validity to the recreation and leisure facet (Skevington and McCrate, 2012). More importantly, the longitudinal data show that significant changes in scores associated with uptake and cessation of participation in community assets demonstrate that these facet scores are highly responsive and sensitive to social change (see also Skevington and Epton, 2018).

When considering the effects of stopping participation in community assets from propensity scores matched with similar individuals who always participated, all four QoL domains reduced during cessation, with the biggest reduction for environmental QoL followed by physical, social then psychological QoL. Each facet also behaved as expected, but the biggest decrease from ceasing to participate was from perceived access to recreation and leisure, which is most pertinent to our case.

There are implications from these findings for each domain, although the causal rationale remains to be further tested. Positive environmental perceptions of QoL may facilitate leaving home to attend community events, as older people believe that for instance, it is physically safe to go out. This is particularly relevant in deprived areas like Salford, UK, where the survey was conducted. Better QoL from social relationships could serve to actively promote communications during participation, as participants are positive about interacting with others. Improvements to QoL associated with mental health may well enhance encounters with others at community events, as happy, confident participants are often more attractive. For the physical domain, features like having more energy should improve the motivation and capacity to leave home, to physically attend the venue, and to be physically active during participation.

Detecting so many important differences between participation groups in this study was only possible due to the many dimensions available in the international WHOQOL-BREF assessment. These results emerge not just from the physical and psychological domains that conventionally respond to participation in community assets, but also from assessing unusual social and environmental QoL domains. Without the WHOQOL-BREF, the latter two outcomes would have been obscured and overlooked, as these changes would not have been captured by measures routinely used for evaluating trials, like the EQ5D.

Using the WHOQOL was advantageous as it detected simultaneous multiple score increases associated with the uptake and cessation of community assets. Furthermore, the positive orientation of WHOQOL-BREF questioning moves the field towards offering a less problem-centred perspective in QoL assessment. At a pragmatic level, tools with an exclusively negative orientation can be depressing for respondents to complete. The positive approach of the WHOQOL is evident from item phrasing, concept labelling, and its rating scales that were developed to assess the very upper end of wellbeing together with the lower. Examining ‘opportunities’ for recreation and leisure illustrates that participation is valued, as part of a positive QoL. Together with opportunities to access information and skills, and to gain health and social care, these three facets resonate with the capabilities approach to QoL proposed by Sen (1994). Furthermore, among international generic QoL measures of this type, leisure is uniquely included in the WHOQOL, and shows the strongest results of all facets to support increase at uptake and decrease at cessation, among all other dimensions tested.

### 4.1 Strengths and limitations

The current study has a number of strengths. First, the longitudinal nature of the data, coupled with a large sample size, allowed us to implement matching methods that reduced the likelihood of potential erroneous conclusions, which may result from cross-sectional studies. Second, rich socioeconomic and demographic information gathered, coupled with links to administrative health records, meant that matching was greatly improved, so we can be more confident that observed differences in QoL cannot be attributed to differences in health state or healthcare utilization, as we can match these. Third, the WHOQOL-BREF measure is attractive for self-completion, as it was co-developed with users of all age groups with wide-ranging sociodemographic features. It is written in comprehensible language, and organised in response scale blocks for speedy completion. With 100 language versions of the WHOQOL-BREF available, our research could be readily replicated worldwide.

Despite visible strengths, there are some potential limitations. First, the study population is from one city in north-west England. However, Salford is consistently ranked amongst the most deprived areas in UK, and hence schemes that work here are likely to be effective in more affluent areas. As living in deprived areas is associated with increased levels of social exclusion (Prattley et al., 2019), specifically targeting these deprived areas will likely lead to higher returns in terms of increased quality of life. Second, the study population comprises older adults, with a number of the older old (>80 years) in the sample. Again however, social prescribing and community assets are designed to reduce loneliness which is most prevalent among the elderly age band (The Kings Fund, 2017).

Another potential limitation is that we did not observe the timing of events. For example, in the cessation analysis we know that individuals ceased participation in community assets and their QoL declined. We assume that the former caused the latter, but it is possible that declining QoL led to a cessation in asset participation. The statistical matching on baseline characteristics should somewhat mitigate against this, if we assume that initial levels of QoL and health indicate similar rates of decline, conditional on age, and other factors. However, without detailed dates of when community asset participation stopped, we cannot be truly certain.

## 5 Conclusions

Our study shows that championing the establishment of community assets can have a widespread effect on the QoL and subjective health of older adults who participate. The findings from longitudinal data affirm that an individual's situation can be improved if they start to participate in community assets. Our evaluation research shows that this type of non-clinical service adds significant value to the range of clinical services currently supplied to older people. Furthermore, in this context, the WHOQOL-BREF is a highly suitable instrument to sensitively assess such complex social interventions. Although widely used to assess interventions, to our knowledge, social prescribing has not been evaluated by this measure before (Skevington and Epton, 2018). It seems plausible that in the absence of appropriate evaluative data, the collective resources of communities may have been previously undervalued. Our study shows that community assets are effective in providing improvements to QoL and subjective health among older adults who are active. More importantly the results highlight the need to maintain engagement in community assets once the decision to participate is taken, as it benefits their QoL and health. Subsequent cessation is associated with some reduction in QoL after stopping, but residual benefits are retained.

This social prescribing approach offers a positive orientation to monitoring QoL in an age group who commonly expect their health and QoL to decline over time. While we show that participants in community asset events do have better lives, we do not know whether this could translate into expectations of a longer life, and this is an area for future research. However, for older populations living in Western countries, improving life quality through promoting active ageing rather than lengthening it, is seen by WHO as a valuable policy goal for modern Western healthcare (WHO, 2002).

In an age of austerity where there is simultaneously an increased demand on formal healthcare services and a reduction in budgets for community services, these findings have useful implications for the provision of resources for assets in the community, and support action against threats to close public amenities, e.g. swimming bath, libraries, village shops, community centres. This evidently damages QoL for people in later life. Facilities supplying community assets are not a luxury, but essential to supplementing the community gaps in formal health and social care provision, so reducing the burden on formal services. Given projections of rising numbers of ageing adults in the foreseeable future, health and social care services must continue to promote community asset use, and maintain these facilities in their local communities, to pre-empt serious consequences to wellbeing that would arise from closure.

## Credit author statement

**Luke Munford:** Conceptualization, Methodology, Validation, Formal Analysis, Writing- Original draft preparation, Writing - Review & Editing, Funding acquisition. **Maria Panagioti:** Conceptualization, Validation, Writing- Original draft preparation. **Pete Bower:** Conceptualization, Resources, Writing- Original draft preparation, Writing - Review & Editing, Supervision, Funding acquisition. **Suzanne Skevington:** Conceptualization, Validation, Resources, Writing- Original draft preparation, Writing - Review & Editing, Supervision.

## Ethics approval and consent to participate

Ethics approval was obtained from the National Research Ethics Service (NRES) North West Lancaster (Research Ethics Committee reference 14/NW/0206).

## Declaration of competing interest

None of the authors have any competing interests to declare.
