# Supplementary material for: Community asset participation and social medicine increases qualities of life
Source: Soc Sci Med. 2020 Aug;259:113149. doi: 10.1016/j.socscimed.2020.113149 (PMC7397510; doi:10.1016/j.socscimed.2020.113149)
Supplement: Multimedia component 2 [file mmc2.docx]

**Supplementary Appendix**

**Figure A1:** A flowchart explaining sample sizes and reasons for losing data from mail out to baseline analysis

12,989

Questionnaires mailed

4,447 (34.2%)

Questionnaires returned

4,377 (33.6%) [100%]

Usable questionnaires

3,686 (28.4%)[84.2%]

Estimation Sample

70 (0.5%)

Excluded as duplicates/not

uniquely identifiable

691 (5.3%) [15.8%]

Excluded as information missing on either community assets participation (667), demographics (18), and/or WHOQOL-BREF (6)

**Figure A2:** A flowchart explaining sample sizes and reasons for losing data from baseline analysis to longitudinal analysis

4,377 (100%) usable questionnaires at baseline

3,390 (77.4%) [100%]

Questionnaires returned

2820 (64.4%)[83.2%]

Estimation Sample

570 (13%) [16.8%]

Excluded as information missing on either community assets participation (534) and/or WHOQOL-BREF (36)

152 (3.5%) did not provide address for follow-up

26 (0.6%) died

809 (18.5%) other attrition

**Table A1:** Balancing of covariates before and after matching

|  | **Uptake Analysis** | | | | **Cessation Analysis** | | | |
| --- | --- | --- | --- | --- | --- | --- | --- | --- |
|  | Treatment  Group | Control Group | | p-value of difference | Treatment  Group | Control Group | | p-value of difference |
|  |  | before | after | after (before) matching |  | before | after | after (before) matching |
|  |  |  |  |  |  |  |  |  |
| EQ5D score | 0.744 | 0.716 | 0.758 | p=0.489 (p=0.127) | 0.749 | 0.799 | 0.754 | p=0.846 (p=0.002) |
| Physical domain | 58.919 | 61.496 | 59.136 | p=0.893 (p=0.160) | 61.915 | 66.849 | 61.849 | p=0.977 (p=0.002) |
| Psychological domain | 70.595 | 68.804 | 71.670 | p=0.447 (p=0.162) | 70.750 | 75.006 | 71.858 | p=0.532 (p<0.001) |
| Social relations domain | 66.422 | 68.644 | 67.406 | p=0.493 (p=0.204) | 68.922 | 72.205 | 68.211 | p=0.760 (p=0.031) |
| Environmental domain | 72.334 | 70.951 | 73.165 | p=0.523 (p=0.225) | 75.433 | 78.519 | 75.386 | p=0.008 (p=0.977) |
| MHI score | 66.353 | 67.788 | 65.710 | p=0.731 (p=0.369) | 66.905 | 72.436 | 64.143 | p=0.244 (p=0.001) |
| *Demographics* |  |  |  |  |  |  |  |  |
| Aged 65-69 years |  |  |  |  |  |  |  |  |
| Aged 70-74 years | 0.259 | 0.273 | 0.259 | p=0.999 (p=0.663) | 0.241 | 0.290 | 0.218 | p=0.186 (p=0.607) |
| Aged 75-79 years | 0.169 | 0.197 | 0.165 | p=0.906 (p=0.305) | 0.206 | 0.235 | 0.216 | p=0.757 (p=0.515) |
| Aged 80-84 years | 0.122 | 0.121 | 0.145 | p=0.435 (p=0.982) | 0.112 | 0.117 | 0.118 | p=0.865 (p=0.834) |
| Aged 85+ years | 0.067 | 0.078 | 0.051 | p=0.453 (p=0.563) | 0.106 | 0.062 | 0.100 | p=0.859 (p=0.032) |
| Female | 0.459 | 0.388 | 0.491 | p=0.365 (p=0.107) | 0.412 | 0.512 | 0.429 | p=0.743 (p=0.015) |
| Live alone |  |  |  |  |  |  |  |  |
| Live with spouse | 0.631 | 0.686 | 0.606 | p=0.472 (p=0.192) | 0.665 | 0.612 | 0.682 | p=0.730 (p=0.184) |
| Live with other | 0.125 | 0.118 | 0.115 | p=0.787 (p=0.654) | 0.071 | 0.101 | 0.041 | p=0.239 (p=0.214) |
| School level education | 0.224 | 0.159 | 0.235 | p=0.753 (p=0.016) | 0.259 | 0.381 | 0.271 | p=0.806 (p=0.002) |
| College level education | 0.071 | 0.042 | 0.075 | p=0.865 (p=0.066) | 0.076 | 0.155 | 0.094 | p=0.562 (p=0.007) |
| University level education | 0.067 | 0.040 | 0.047 | p=0.340 (p=0.075) | 0.053 | 0.109 | 0.041 | p=0.610 (p=0.025) |
| NVQ level education | 0.255 | 0.253 | 0.278 | p=0.549 (p=0.942) | 0.324 | 0.261 | 0.394 | p=0.176 (p=0.084) |
| Professional education | 0.200 | 0.147 | 0.180 | p=0.574 (p=0.042) | 0.194 | 0.277 | 0.194 | p=0.999 (p=0.022) |

**Table A2:** Determinants of attrition (including mortality)

|  | Main effect | | | Interaction with Baseline CA participation | | |
| --- | --- | --- | --- | --- | --- | --- |
|  | Odds ratio | p-value | 95% CI | Odds ratio | p-value | 95% CI |
| Physical domain | 1.005 | 0.340 | [0.995 to 1.014] | 1.002 | 0.835 | [0.987 to 1.017] |
| Psychological domain | 0.991 | 0.080 | [0.982 to 1.001] | 0.998 | 0.772 | [0.982 to 1.013] |
| Social relations domain | 1.000 | 0.914 | [0.993 to 1.007] | 0.999 | 0.846 | [0.988 to 1.010] |
| Environmental domain | 0.999 | 0.869 | [0.989 to 1.009] | 0.992 | 0.323 | [0.977 to 1.008] |
| **EQ5D Health Utility Index** | **0.461*** | **0.047** | **[0.215 to 0.989]** | 1.341 | 0.655 | [0.371 to 4.850] |
| Participate in CAs as baseline | 1.116 | 0.848 | [0.363 to 3.429] |  |  |  |
| Male | Reference |  |  |  |  |  |
| Female | 0.933 | 0.577 | [0.733 to 1.189] | 1.279 | 0.188 | [0.887 to 1.844] |
| Age 65 - 69 | Reference |  |  |  |  |  |
| Age 70 - 74 | 1.185 | 0.265 | [0.879 to 1.596] | 1.083 | 0.733 | [0.686 to 1.709] |
| **Age 75 - 79** | **1.392*** | **0.042** | **[1.012 to 1.915]** | 1.009 | 0.973 | [0.622 to 1.636] |
| **Age 80 - 84** | **1.517*** | **0.026** | **[1.051 to 2.188]** | 0.947 | 0.852 | [0.534 to 1.678] |
| **Age 85 - 98** | **2.211***** | **<0.001** | **[1.467 to 3.334]** | 1.143 | 0.677 | [0.610 to 2.139] |
| Live alone | Reference |  |  |  |  |  |
| Live with spouse | 1.073 | 0.566 | [0.844 to 1.364] | 0.963 | 0.839 | [0.672 to 1.381] |
| Live with other | 1.368 | 0.053 | [0.996 to 1.878] | 0.914 | 0.728 | [0.551 to 1.517] |
| No qualifications | Reference |  |  |  |  |  |
| School level Qualifications | 0.924 | 0.644 | [0.662 to 1.291] | 0.634 | 0.055 | [0.399 to 1.009] |
| College level Qualifications | 0.738 | 0.362 | [0.384 to 1.418] | 0.767 | 0.546 | [0.323 to 1.818] |
| University level Qualifications | 0.889 | 0.700 | [0.488 to 1.620] | 1.145 | 0.743 | [0.510 to 2.573] |
| **NVQ and Trade Qualifications** | **0.660**** | **0.003** | **[0.500 to 0.872]** | 1.485 | 0.128 | [0.892 to 2.473] |
| Professional Qualifications | 0.795 | 0.060 | [0.627 to 1.009] | 1.451 | 0.072 | [0.968 to 2.176] |
| *Presence of limiting condition* |  |  |  |  |  |  |
| **Asthma** | **0.481*** | **0.017** | **[0.264 to 0.877]** | 1.176 | 0.772 | [0.392 to 3.525] |
| Cancer | 1.166 | 0.712 | [0.517 to 2.627] | 1.457 | 0.572 | [0.394 to 5.382] |
| Back pain/Sciatica | 0.772 | 0.147 | [0.544 to 1.095] | 0.784 | 0.428 | [0.429 to 1.431] |
| **Bronchitis/COPD** | **1.572*** | **0.047** | **[1.005 to 2.457]** | 1.381 | 0.406 | [0.645 to 2.956] |
| Kidney disease | 1.163 | 0.757 | [0.448 to 3.018] | 1.337 | 0.755 | [0.215 to 8.308] |
| Colon/Irritable bowel | 0.823 | 0.496 | [0.470 to 1.441] | 0.737 | 0.517 | [0.292 to 1.859] |
| Congestive heart failure | 1.158 | 0.711 | [0.533 to 2.517] | 2.374 | 0.158 | [0.715 to 7.884] |
| Diabetes | 0.946 | 0.844 | [0.542 to 1.650] | 1.220 | 0.669 | [0.491 to 3.032] |
| Hard of hearing | 1.261 | 0.205 | [0.881 to 1.806] | 1.148 | 0.634 | [0.650 to 2.030] |
| Heart disease/angina | 0.735 | 0.190 | [0.464 to 1.165] | 0.948 | 0.898 | [0.419 to 2.147] |
| High blood pressure | 1.142 | 0.597 | [0.699 to 1.865] | 0.970 | 0.944 | [0.410 to 2.292] |
| High cholesterol | 0.991 | 0.976 | [0.569 to 1.726] | 1.258 | 0.648 | [0.470 to 3.368] |
| Osteoarthritis | 0.992 | 0.963 | [0.701 to 1.403] | 1.043 | 0.884 | [0.590 to 1.844] |
| **Osteoporosis** | **1.742*** | **0.030** | **[1.054 to 2.879]** | 0.565 | 0.232 | [0.221 to 1.440] |
| Overweight | 0.891 | 0.638 | [0.551 to 1.440] | 0.744 | 0.475 | [0.331 to 1.673] |
| Poor circulation in legs | 1.412 | 0.056 | [0.991 to 2.012] | 1.065 | 0.837 | [0.585 to 1.937] |
| Rheumatoid arthritis | 1.044 | 0.840 | [0.686 to 1.590] | 0.682 | 0.374 | [0.294 to 1.583] |
| Rheumatic disease | 1.552 | 0.291 | [0.686 to 3.514] | 0.510 | 0.467 | [0.083 to 3.133] |
| Stomach problem/ulcer/etc· | 0.972 | 0.911 | [0.588 to 1.606] | 0.721 | 0.447 | [0.311 to 1.673] |
| Stroke | 1.395 | 0.331 | [0.713 to 2.726] | 0.434 | 0.160 | [0.135 to 1.391] |
| Thyroid disorder | 0.974 | 0.943 | [0.466 to 2.036] | 1.540 | 0.440 | [0.514 to 4.613] |
| Problems with vision | 0.998 | 0.991 | [0.659 to 1.510] | 0.814 | 0.555 | [0.411 to 1.613] |
| Other conditions | 1.329 | 0.306 | [0.771 to 2.289] | 1.068 | 0.877 | [0.465 to 2.451] |

Bold indicates statistical significance at p<0.05. * p<0.05; ** p<0.01; *** p<0.001
